# Dynamics of Antibiotic Resistant *Mycobacterium tuberculosis* during Long-Term Infection and Antibiotic Treatment

**DOI:** 10.1371/journal.pone.0021147

**Published:** 2011-06-16

**Authors:** Solomon H. Mariam, Jim Werngren, Joakim Aronsson, Sven Hoffner, Dan I. Andersson

**Affiliations:** 1 Swedish Institute for Infectious Disease Control, Solna, Sweden; 2 Aklilu Lemma Institute of Pathobiology, Addis Ababa University, Addis Ababa, Ethiopia; 3 Department of Infectious Diseases, County Hospital Ryhov, Jönköping, Sweden; 4 Department of Medical Biochemistry and Microbiology, Uppsala University, Uppsala, Sweden; Swiss Tropical and Public Health Institute, Switzerland

## Abstract

For an infecting bacterium the human body provides several potential ecological niches with both internally (e.g. host immunity) and externally (e.g. antibiotic use) imposed growth restrictions that are expected to drive adaptive evolution in the bacterium, including the development of antibiotic resistance. To determine the extent and pattern of heterogeneity generated in a bacterial population during long-term antibiotic treatment, we examined in a monoclonal *Mycobacterium tuberculosis* infection antibiotic resistant mutants isolated from one patient during a 9-years period. There was a progressive accumulation of resistance mutations in the infecting clone. Furthermore, apparent clonal sweeps as well as co-existence of different resistant mutants were observed during this time, demonstrating that during treatment there is a high degree of dynamics in the bacterial population. These findings have important implications for diagnostics and treatment of drug resistant tuberculosis infections.

## Introduction

In 2009, tuberculosis disease (TB) due to *Mycobacterium tuberculosis* infections caused an estimated 1.3 million deaths globally [1]. When active TB is manifested the infection is normally treated with antibiotics, thereby providing an external pressure for the selection of antibiotic resistant bacilli. The risk of resistance development will be determined by a number of different factors, including the antibiotic selective pressure (set by the number, dosing and quality of the used drugs), any pre-existing resistances in the infecting clone, the immune status of the treated individuals and their compliance with the drug regime [2]–[4]. In addition, since all resistance development in *M. tuberculosis* occurs via mutational changes in the chromosome, the bacterial population size and the bacterial mutation rate will also affect the probability by which resistant clones appear *de novo* during treatment [5]. Furthermore, the ability of a resistant clone to grow and rise in frequency in the treated individual is influenced by the effect of the resistance on bacterial fitness [6], [7].

The primary drugs used in treatment of *M. tuberculosis* infections are isoniazid and rifampicin and resistance to these drugs may be caused by mutations in a number of genes, including *inhA, katG*, and *ndh*, (isoniazid) and *rpoB* (rifampicin) [8]–[11]. Other primary drugs used include ethambutol, pyrazinamide and streptomycin where mutations in the *embB* (ethambutol), *pncA* (pyrazinamide) and *rpsL/rrs* (streptomycin) genes confer resistance [12]–[14]. In addition, there are several second-line antibiotics available but many of them are less efficacious, give more side-effects and are more expensive than the first-line drugs. Examples of second-line drugs include aminoglycosides, polypeptides, fluoroquinolones, thioamides, cycloserine and p-aminosalicylic acid [15].

The effects of resistance to rifampicin, isoniazid and streptomycin on fitness have been examined in *M. tuberculosis* and other bacterial species, and depending on which gene is mutated and the particular mutation the effect might range from no effect to a severe reduction in fitness. For example, most mutations in *rpoB* seem to reduce fitness [16]–[19]. In contrast, the fitness effects of mutations in the *rpsL* and *katG* genes vary greatly depending on the specific mutation. Thus, the *katG* mutation Ser_315_→Thr and *rpsL* mutation Lys_42_→Arg have no or small effects on fitness, while the Thr_275_→Pro (*katG*) and Lys_42_→Thr and Lys_42_→Asn (*rpsL*) mutations significantly reduce fitness [20]–[26]. As expected, the frequencies by which these different mutants are recovered clinically from patients correlate with their fitness measured *in vitro* [17], [24]–[26]. These findings demonstrate that fitness is a major determinant of the ability of these different resistant mutants to spread and establish infections [7], [25].

Even though several studies of the long-term dynamics of drug resistance in rapidly mutating viruses with large populations have been performed [27], [28], less is known about resistance dynamics in bacteria. Partly this is because the lower mutation rates in bacteria, and the resulting lower rates by which drug resistant variants appear, imply that there is less dynamics in the population. However, some studies of bacterial diversity in, for example, *Pseudomonas aeruginosa* [29], [30] and *Helicobacter pylori* [31], [32] suggest that also in bacterial infections population heterogeneity might be considerable, especially during long-term or chronic infections.

To address the extent of heterogeneity and dynamics in antibiotic resistant variants during a monoclonal infection in a host, we examined serial *M. tuberculosis* Beijing isolates obtained from one patient during a 9-year period of infection and disease where the successive accumulation of resistance mutations ultimately made the infection untreatable and caused death of the patient. Her isolates belonged to the W-Beijing family of *M. tuberculosis*, which are especially prevalent in East Asia and Eastern Europe [33]–[35], and are genetically identified by their characteristic IS*6110* and spoligotypes patterns and *katG*463and *gyrA*95 mutations [35]. Moreover, we examined the heterogeneity and dynamics with respect to aminoglycoside resistance within 10 single colony isolates (sub-clones) derived from each of the original serial patient isolates. Analysis of these sub-clones showed that there is a high degree of dynamics in the infecting bacterial population, including successive changes in antibiotic resistance types and bacterial fitness. These findings have important implications for treatment and diagnostics of antibiotic resistance in infected individuals.

## Materials and Methods

### Isolates

The isolates that were characterized were clinical isolates obtained from the same patient during a period of 9 years (1991–1999). These isolates were kept frozen at −70°C in Middlebrook 7H9 broth containing 10% glycerol. A fresh aliquot was taken for analysis and prior to all experiments, the isolates were cultured on Lowenstein-Jensen slants for 3 to 4 weeks at 37°C. The isolates and the time of isolation together with their drug resistance patterns are listed in Table 1.

**Table 1:** Date of isolation and resistance pattern of the different isolates of *M. tuberculosis* as tested in the BACTEC 460 system and antibiotic resistance mutations identified in isolates obtained from eight different time points.

IPatient isolate/date isolated(D/M/Y)/isolate No.	Drug/target gene sequenced									
	Isoniazid/*katG*		Rifampicin/*rpoB*		Streptomycin/*rpsL*		Ethambutol/*embB*		Amikacin/*rrs* b	
	S/R^a^	Mutation	S/R^a^	Mutation	S/R^a^	Mutation	S/R^a^	Mutation	S/R^a^	Mutation
1/2-4-91/S91-222	R	AGC315ACC	S	wt	S	wt	R	wt	S	wt
2/12-7-91/S91-224	R	AGC315ACC	R	GAC516GTC	S	wt	R	wt	S	wt
3/11-9-91/S91-263	R	AGC315ACC	R	GAC516GTC	R	AAG87AGG	R	wt	S	wt
4/13-12-91/S92-001	R	AGC315ACC	R	GAC516GTC	R	wt	R	wt	S	A513C
5/5-2-92/S92-031	R	AGC315ACC	R	GAC516GTC	R	AAG87AGG	R	wt	S	wt
6/14-12-92/S93-007	R	AGC315ACC	R	GAC516GTC	R	AAG42AGG	R	wt	S	wt
7/5-2-93/S93-021	R	AGC315ACC	R	GAC516GTC	R	wt	R	wt	S	A513C
8/N-3-99/S99-293	R	AGC315ACC	R	GAC516GTC	R	AAG42AGG	R	wt	R	A1400G

a : S = Susceptible, R = Resistant.

wt = wild-type.

b : Streptomycin-resistant isolates with wild-type *rpsL* were checked for mutation in *rrs*. The A513C mutation in *rrs* confers streptomycin resistance whereas the A1400G mutation confers amikacin resistance.

### Drug Susceptibility Testing of Serial Isolates

Drug susceptibility testing of the isolates to the various drugs (isoniazid, rifampicin, streptomycin, ethambutol, amikacin) were performed using the BACTEC 460TB system according to the instructions of the manufacturer.

### DNA Extraction

For DNA extraction, the isolates were first grown on Lowenstein-Jensen (LJ) medium. Bacterial cells were then transferred into 1.5 ml micro-centrifuge tubes containing 250 µl of 1× TE buffer using a sterile loop followed by killing by heating at 80°C for 1 hr. Cells were then centrifuged at 13,000 rpm for 2 min. The supernatant was discarded and the pellet resuspended in 500 µl of 150 mM NaCl, followed by centrifugation. This was repeated once. Finally, the DNA pellet was resuspended in 25 µl of TE buffer.

### Amplification of DNA by PCR and Sequencing

PCR reactions were carried out in a 50 µl volume containing 0.5 µM of each primer, 200 µM of each dNTP, 5 µl of 10× PCR buffer, 0.25 µl of AmpliTaq Gold DNA polymerase, 1 µl of DNA and 2 mM MgCl_2_ except for the primer pair used to amplify the 1400 region of *rrs*, for which the MgCl_2_ concentration was 3 mM. The primers, size of fragments targeted for amplification and cycling conditions are listed in Table S1. The GeneAmp® PCR System 9700 was used for running PCR and sequencing reactions. PCR products were purified using GFX™ PCR and Gel Band Purification Kit (Amersham Pharmacia Biotech). The same primers as used for PCR were used in cycle sequencing reactions. The sequenced genes were *katG*, *rpoB*, *rpsL*, *embB* and *rrs*. Sequencing was performed using both forward and reverse primers. Sequencing reactions were run with an initial denaturation at 96°C for 1 min followed by 25 cycles each at 96°C for 10 sec, 50°C for 5 sec and 60°C for 4 min. Sequences were obtained using ABI PRISM 3100 Genetic Analyzer. Mutations were identified by importing sequencing products into the BLAST2 nucleotide sequence alignment tool.

### Analysis of aminoglycoside resistance mutation spectra in cub-clones

zzzzBecause initial sequencing of the gene (*rpsL* and *rrs*) segments in which mutations occur to cause streptomycin and amikacin resistance in the successive serial isolates revealed changing patterns (wild-type to mutant and back to wild-type again), each of the 8 isolates were first grown on duplicate plates on Middlebrook 7H10 medium containing 10% OADC enrichment (M7H10 medium) to obtain single colonies. Then, 10 single colonies (sub-clones) were randomly picked from two different plates on day 16 of culture and grown in Middlebrook 7H9 medium containing 10% ADC enrichment and 0.1% Tween 80 (M7H9 medium) for 3 weeks followed by inoculation onto LJ medium. DNA extraction, PCR amplification and sequencing were performed as described above for the isolates to identify the spectrum of mutations.

### Molecular Typing of Serial Isolates

The serial isolates were typed by both spoligotyping [36] and restriction fragment length polymorphism (RFLP) [37]. The similarities of the RFLP patterns were determined by using BioNumerics version 3.0 software (Applied Maths, Sint Maarten Latem, Belgium). Results are shown in Figure S1.

### Determination of relative fitness by competition experiments

To determine the relative fitness of the various isolates, head-to-head competitions were performed in liquid cultures. Isolate 1.1 (isolated on 2/4/91 from the patient) was arbitrarily chosen as the parent and all competitions were then performed between this parental isolate and sub-clones from subsequent isolates. Since the parent isolate was susceptible to rifampicin and all the subsequent isolates were highly resistant, rifampicin was used to distinguish the parent and competitors. The parental isolate and the competitors were inoculated from Lowenstein-Jensen slants and grown separately in 7H9 medium containing 0.1% Tween 80 for one week prior to use. After homogenization of the cultures by sonication for 5 min, optical densities of the parental and competitors were adjusted to the same value (A_600_ = 0.1) and then 10 µl of the parental and 30 µl of the competitor bacterial suspensions were mixed in 20 ml of 7H9 medium. Since we expected that the mutants would be outcompeted, competition experiments were started with mutants in slight excess (3∶1) to increase the range where the mutants could be followed. Immediately after mixing, dilutions were prepared from each mixed culture and plated in duplicate on both drug-free and rifampicin-containing (2 ug/ml) M7H10 plates. Cultures were incubated in M7H9 medium at 37°C with regular shaking and the plating procedure was then repeated after 14 days of growth. The number of colony forming units (CFUs) on the agar plates was counted after 25 days of incubation at 37°C. The CFU counts of the wild type were obtained by subtracting the CFU on drug-containing plates from the total CFU on drug-free plates. The competition index (CI) was calculated as the ratio of CFU (mutant)_14 days_/CFU (parental)_14 days_ divided by CFU (mutant)_0 days_/CFU (parental)_0 days_. A CI value >1 indicates that the competitor outcompeted the parent and <1 the opposite. Fourteen days of incubation correspond to approximately 15–20 generations of growth for the parental strain. The data presented represent the averages of two independent experiments for each of the mutant types. Control experiments showed that the plating efficiency of the resistant mutants was unaffected by the presence of rifampicin.

### Ethics Statement

This *in vitro* study was carried out on a number of clinical isolates of *M. tuberculosis* obtained from one patient with multi-drug-resistant tuberculosis. The isolates were collected during the period 1991 to 1999 and kept at the strain collection of the Swedish Institute for Infectious Disease Control. The patient died in 2000, four years before the study was initiated, and the results obtained did not in any way affect the clinical treatment or outcome, i.e. the samples were taken as part of routine clinical care and specific consent was therefore not needed and acquired. Exemption from ethical approval was provided by the institutional review board at the Karolinska Institute (Regional Ethical Review Board in Stockholm, FE 289 171 77 Stockholm) who stated that this *in vitro* study on clinical isolates and their drug resistance carried out at our laboratory is excluded from the demand of a specific ethical permission.

## Results

### Patient data, antibiotic prescription and antibiotic resistance from 1991–1999

At the time of diagnosis of lung tuberculosis, March 1991, the Swedish female patient was 19 years old and she was then intermittently antibiotic treated until she died of tuberculosis in July 2000. The treatment and resistance pattern is shown in Table 2 and indicates the types of antibiotic used during the period 1991–2000 and the resistance pattern of the bacteria. Standard drug susceptibility testing [using the BACTEC 460TB system of the serially obtained bacterial samples showed that the isolates exhibited resistance to an increasing number of drugs (Table 1). The first isolate was resistant to isoniazid only. The second isolate also acquired resistance to rifampicin. The third through seventh isolates were resistant to isoniazid, rifampicin and streptomycin and this triple resistance developed within about six months (Table 1). The eighth isolate was resistant to all these drugs and to the second-line drug amikacin.

**Table 2:** Antibiotic resistance and prescription pattern during 1991–2000.

Date	Apr91	May91	May91	Jul91	Jul91	Aug91	Nov91	Dec91	Dec91	Apr92	Oct92	Oct92	Aug93	Oct93	Nov93	Oct93	Nov93	Dec93	Mar94	Mar94	Jul94	Jul94	Sep94	Sep94	Oct94	Nov94	Feb95	Mar95	Jun95	Sep95	Dec95	Dec95	Apr99	Apr99	Jun99	Jun99	Jul99	Sep99	Sep99	Jan00	Jan00
**Antibiotics**																																									
Azithromycin																																					R		R		
Amikacin								X			X		X	R	R		R		R	R	R	R	R	R		R	R	R	R	R	R		R				R		R	R	
Capreomycin														I	S	X	S	X	I		S	S	S	R	X	S	S	S	I	I	I		R				I		S		X
Ciprofloxacin				X		X		X	R	R	X	R	X	R	S	X	S	X	S	S	S	S	S	S	X	S	S	S	S	S	S	X	S	X	X	X	S		S	S	X
Clarithromycin							R		R	R		R			R																		R			X	S	X	R	S	X
Clofazimin								X		R	X		X	S	S	X	S	X	S		S	S	S	S	X	S	S	S	S	S	S		S	X	X	X	S	X	S		X
Cycloserine									R	R		R																									R			S	
Ethambutol		S	X	X	R	X	R		R	R		R		I	R	X	I	X	I	S	I	S	S	R	X	R	S	R	R	R	R	X	R	X	X	X		X	R	S	X
Ethionamide								X			X			I	S	X	I	X	I		S	I	I	R	X	I	S	R	R	I	R	X	R	X			I		R		
Isoniazid	X	R			R		R		R	R		R								R			R	R									R	X					R	R	
Levofloxacin																																						X			
PABA							R		R	R		R		R																							R				
Protionamide													X																											S	X
Pyrazinamide	X		X	X		X														S													S	X	X	X		X	S	S	X
Rifabutin											X	R	X	I	R		R		I		I	I	I	R	X	R	I	R	R	R	R	X	R	X					R		
Rifampicin	X	S	X	X		X	R		R	R		R			R					R			R										R						R	R	
Streptomycin		S		S					R	R		R								R			R										R						R	R	
Thioacetazone														R	R																						R				

An X indicates that the patient was treated with that antibiotic. R, I and S refer to resistant, intermediate and susceptible. The patient was not treated during the period 13 Nov 1996 to 8 Apr 1999.

### IS*6110* based RFLP and spoligotyping indicates the presence of one clone during infection

The IS*6110* RFLP patterns of the bacterial isolates isolated from 1991 to 1999 were identical as shown in Fig. S1. In addition, the spoligotyping pattern showed that they had the 9-band hybridisation to spacers 35–43 characteristic of Beijing isolates (Fig. S1). These data strongly suggest that the patient carried the same single clonal type during 9 years of infection and that no re-infection with other strains occurred during this period.

### Heterogeneity of resistance mutations between different time points

The respective gene fragments of these eight isolates were subjected to PCR and DNA sequencing to detect the presence of potential mutations in the *katG*, *rpoB*, *rpsL*, *embB* and *rrs* genes. Initial sequencing revealed that all eight isolates carried the AGC315→ACC mutation responsible for isoniazid resistance and an additional CGG463→CTG mutation in *katG*. The CGG→CTG mutation at codon 463 has been shown to be prevalent in Beijing family isolates and is not associated with INH resistance [34], [38], [39]. As shown in Table 1, all but the first isolate harboured the GAC516→GTC mutation in the *rpoB* gene. None of the isolates carried any mutations in the *embB* gene but ethambutol resistance was observed (Table 1), implying that ethambutol resistance occurred by an *embB*-independent mechanism [40], [41]. All isolates were susceptible to amikacin except for the last isolate (isolate 8), which carried the A1400→G mutation in *rrs*, which has been shown to be responsible for amikacin resistance in both clinical and *in vitro* isolates of *M. chelonae, M. abscessus* and *M. tuberculosis* [42], [43].

### Dynamics of streptomycin resistance mutations within and between sub-clones

The initial sequencing of all isolates showed the presence of one or the other of at least three different mutations in *rpsL* or *rrs*. These were the AAG87→AGG and AAG42→AGG mutations in *rpsL*, and A513→C in *rrs* in isolates 3–8. These data thus suggested that there might be heterogeneity with regard to the streptomycin resistance mutations within the bacterial population isolated from each time point (note that the initial testing of drug susceptibility and sequencing of the *rpsL* and *rrs* genes were performed on bacterial populations obtained from each time point and that had not been single-cell colony purified). To assess the potential genetic heterogeneity within each population, the *rpsL* and *rrs* gene segments responsible for streptomycin resistance were sequenced from 10 randomly selected sub-clones obtained from the eight time points (Table 3).

**Table 3:** Heterogeneity in types of streptomycin resistance mutations isolated during treatment.

Parent isolate	Sub-clones	Gene		
		*rpsL*(numbers indicate amino acid position)	*rrs*	
			Nucleotide A513	Nucleotide A1400
1 (S 91-222)	1.1–1.10	wt	wt	wt
2 (S 91-224)	2.1–2.10	wt	wt	wt
3 (S 91-263)	3.1,3.3–3.7,3.9–3.10	AAG87→AGG	wt	wt
3 (S 91-263)	3.2	wt	A513→C	wt
3 (S 91-263)	3.8	AAG42→AGG	wt	wt
4 (S 92-001)	4.1–4.2,4.4–4.10	AAG87→AGG	wt	wt
4 (S 92-001)	4.3	AAG42→AGG	wt	wt
5 (S 92-031)	5.1–5.4,5.6,5.8–5.9	AAG87→AGG	wt	wt
5 (S 92-031)	5.5	AAG87→ANG	wt	wt
5 (S 92-031)	5.7	ND	wt	wt
5 (S 92-031)	5.10	wt	wt	wt
6 (S 93-007)	6.1–6.10	AAG42→AGG	wt	wt
7 (S 93-021)	7.1,7.7	AAG42→AGG	wt	wt
7 (S 93-021)	7.2–7.6, 7.8–7.10	wt	A513→C	wt
8 (S 99-293)	8.1–8.7,8.9–8.10	AAG42→AGG	wt	A1400→G
8 (S 99-293)	8.8	wt	wt	A1400→G

From both the susceptibility data and sequencing of the *rpsL* gene, sub-clones of isolates 1 and 2 were shown to be sensitive to streptomycin and wild-type in *rpsL* (Tables 1 and 4). Most sub-clones of subsequent isolates 3, 4 and 5 harbored the AAG87→AGG streptomycin resistance mutation, with only two sub-clones harboring the AAG42AGG mutation in *rpsL* (sub-clones 3.8 and 4.3) or A513C mutation in *rrs* (sub-clone 3.2) (Table 3). (The A513→C mutation alone in *rrs* is known to confer streptomycin resistance in *E. coli* [44] and clinical isolates of *M. tuberculosis* [14]). Thus, the heterogeneity was evident within and between the sub-clones (e.g., 3.2 and 3.8, which are different from each other and the other sub-clones of isolate 3 as well as from the sub-clones of isolate 5, Table 3). A sub-clone of isolate 5 (5.5) yielded ambiguous sequencing result in *rpsL* (AGG87→ANG. One sub-clone (5.10) was found to be wild-type for both *rpsL* and *rrs*.

**Table 4:** Relative fitness expressed as competition indexes (CI) and frequency of different resistant mutants isolated at the different time points.

Time	(isolate)	CI a	Type	Frequency b
1991	(1, parent)	1 (0.7, 1.3)	wt	10/10
1991	(3.1)	0.3 (0.3, 0.3)	*rpsL*87AGG	9/10
1991	(3.2)	<0.01 (<0.01, <0.01)	*rrs*A513C	1/10
1992	(4.4)	0.3 (0.2, 0.4)	*rpsL*87AGG	10/10
1992	(5)	8 (6, 11)	*rpsL*87AGG	10/10
1993	(6)	0.3 (0.2, 0.4)	*rpsL*42AGG	10/10
1993	(7.1)	0.07 (0.04, 0.1)	*rpsL*42AGG	2/10
1993	(7.2)	1 (1, 1)	*rrs*A513C	8/10
1999	(8)	0.5 (0.3, 0.6)	*rpsL*42AGG, *rrs*A1400G	10/10

CIs were determined as described in Materials and Methods.

a Numbers are averages based on two independent measurements (shown within parantheses).

b Frequency indicates how many of the 10 sub-clones obtained from that particular time point and isolate carried the specified resistance mutation.

With regard to the streptomycin resistance mutations, at least four different types (*rpsL*87AGG, *rrsA*513C, *rpsL*42AGG and *rpsL42*AGG +*rrsA*1400G) were identified during the sampling period. This is likely to be an underestimate since we only examined 10 independent sub-clones for each time point and during the period 1993–1999 sampling was sparse. A shift in the bacterial population from AAG87→AGG to the AAG42→AGG variant is seen from isolate 6 onwards (except for isolate 7 most sub-clones of which carried the A513→C mutation in *rrs*, with only two of its sub-clones [7.1 and 7.7] carrying the AAG42→AGG mutation).

### Fitness and frequencies of different streptomycin resistant types

To determine the competitive fitness of the different streptomycin resistant mutants isolated during the course of the infection, we performed *in vitro* competition experiments where the different streptomycin resistant sub-clones were competed against a sub-clone obtained from the first isolate obtained in 1991 (resistant to isoniazid and susceptible to rifampicin, ethambutol, streptomycin and amikacin, see 1 [S91-222 (1, parent)] in Table 4. *In vitro* competitions in rich culture medium have been shown in previous experiments to provide a relevant measure of the frequency by which the resistant mutants are recovered in clinical settings [17], [24]–[26]. The relative fitness of the first isolate was set to 1.0 and all subsequent isolates were compared to this strain (Table 4). When comparing isolates with same resistance mutation *rpsL*87AGG (i.e. 3.1 and 4.4 with 5) it can be seen that in some cases fitness appeared to increase over time (competition index [CI] increased from 0.3 to 8). Similarly, when comparing the isolates with mutation *rrsA*513C (i.e. 3.2 with 7.2) the competition index was increased from <0.01 to 1, suggesting a substantial increase in fitness. However, there were also exceptions to this trend such that less fit mutants appeared over time (for example, replacement of the fit 1992, rpsL87AGG mutant by the less fit 1993, rpsL87AGG). Thus, we could not observe any general trend that fitness was increased over time.

## Discussion

The presented results show that there is extensive dynamics in the bacterial population during a long-term *M. tuberculosis* infection. As expected, over time and with cumulative antibiotic exposure there was a progressive accumulation of resistances in the infecting clone. More unexpected was the dynamics observed for the streptomycin resistant sub-clones, where successive clonal sweeps as well as transient co-existence of different streptomycin-resistant variants were observed during the 9-year period (Table 3). During growth in the patient fitness of the antibiotic resistant sub-clones changed (Table 4), suggesting that they adapted to the fitness costs associated with the resistance mutations. Thus, in several cases fitness appeared to increase over time but in other cases fitness was reduced, suggesting that the within host dynamics is complex and that the growth rate measured in vitro is not necessarily a direct predictor of bacterial growth and survival within a patient.

It is commonly assumed in bacterial infections that one resistance type (primary or acquired) goes to fixation in the population in response to an imposed antibiotic pressure. Instead of such fixation, we observed successions as well as co-existence of resistant mutants (Table 3). These results are of relevance both from an evolutionary point of view as well as for diagnostics and treatment of TB. As seen from previous *in vitro* fitness assays, several of the common resistance mutations (e.g. *rpsL*87AGG and *rrsA*513C) found in clinical isolates of *M. tuberculosis* cause a reduction in fitness [24]–[26], [45]. These mutations were also found in isolates from this patient. In some certain cases, these resistant mutants either disappeared or were replaced by other more fit mutants (e.g. *rpsL87*AGG was replaced by *rpsL42*AGG from 1992 to 1993) or their fitness was increased due to other unknown mutations (e.g. subclones 3.1 and 4.4 versus 5, and subclones 3.2 versus 7.2) [19], [45], [50]. Thus, it is possible that more fit multi-resistant mutants may be selected during treatment and subsequently spread to other individuals [6], [25]. Interestingly, the succession of streptomycin resistant *rpsL* mutants (i.e. *rpsL87*AGG was replaced by *rpsL42*AGG) observed in our isolates is reminiscent of that seen in a previous study where serial isolates from a single patient showed a similar succession of *rpsL* mutant types [46]. Furthermore, heterogeneity in resistance types can involve other drugs than streptomycin, as shown for ethambutol and fluoroquinolones where parallel evolution in distinct lung compartments from a single founder strain was suggested to generate heterogeneity in resistance-associated alleles for isolates with identical IS*6110* RFLP pattern [47].

It has been shown that heterogeneity exists in the resistant bacterial populations within the host [46], [47] and it is conceivably that the frequencies with which different drug resistant mutants are isolated in patients partly is determined by their differences in fitness. Support for this notion comes from the observation that a positive correlation between the frequency of isolation of resistant mutants and their relative fitness (e.g., Ser531→Leu in *rpoB* (rifampicin resistance), Lys42→Arg in *rpsL* (streptomycin resistance), Ser315→Thr in *katG* (isoniazid resistance) has been reported for *M. tuberculosis* [5], [17], [19], [23]–[26]. There is ample support for compensatory evolution of fitness costs associated with resistance mutations [16], [20]–[21], [45]–[52], and it is conceivable that such evolution also influences the fitness of the resistant mutants studied in this and other work. Thus, the differences in fitness observed between sub-clones with the same resistance mutations might indicate compensatory evolution. However, we cannot exclude the possibility that the observed fitness differences are caused by other fitness-altering genetic changes, unrelated to the resistance mutations.

Finally, our findings are relevant from the point of view of resistance diagnostics. Thus, most current clinical and research procedures to determine resistance patterns are based on examination of single isolates from a given disease episode with the assumption that this isolate is representative of a homogenous bacterial population. Recent studies have shown that this assumption is invalid and demonstrated the occurrence of both mixed clonal infections as well as exogenous re-infection where a patient is re-infected by a second clone during a recurrent episode [53], [54]. This study demonstrates that within a specific clone there is also extensive heterogeneity with respect to the resistant mutants selected and maintained over time. Since fitness and resistance levels might vary between resistant types it is important to know the population composition at different time points to assure that the proper drug combinations are used. To obtain a measure of this heterogeneity several independent bacterial clones needs to be examined from each patient sample. Furthermore, the heterogeneity in resistance mutations observed here is most likely an underestimate since we only examined streptomycin resistance, sampling was sparse during some time intervals (i.e. 1993–1999), and only ten sub-clones were studied from each sampling.

## Supporting Information

Figure S1 Spoligotyping and RFLP pattern of serial clinical isolates of *M. tuberculosis*. S99-293 = isolate 8, S91-222 = isolate 1, S91-224 = isolate 2, S91-263 = isolate 3, S92-001 = isolate 4, S92-031 = isolate 5, S93-007 = isolate 6, S93-021 = isolate 7.
(DOCX)

Table S1 Primers and PCR conditions for amplification of resistance genes.
(DOCX)

## References

[1] World Health Organization (2009). WHO Report 2009. Global tuberculosis control, surveillance, planning, financing.. A Short Update to the 2009 Report.

[2] Austin DJ, Kristinsson KG, Anderson RM (1999). The relationship between the volume of antimicrobial consumption in human communities and the frequency of resistance.. Proc Natl Acad Sci U S A.

[3] Lipsitch M, Samore MH (2002). Antimicrobial use and antimicrobial resistance: a population perspective.. Emerg Infect Dis.

[4] Handel A, Margolis E, Levin BR (2008). Exploring the role of the immune response in preventing antibiotic resistance.. J Theor Biol.

[5] Gillespie SH (2002). Evolution of drug resistance in *Mycobacterium tuberculosis*: clinical and molecular perspective.. Antimicrob Agents Chemother.

[6] Cohen T, Murray M (2004). Modeling epidemics of multidrug-resistant *M. tuberculosis* of heterogeneous fitness.. Nat Med.

[7] Andersson DI, Hughes D (2010). Antibiotic resistance and its costs: is it possible to reverse resistance?. Nat Rev Microbiol.

[8] Zhang Y, Heym B, Allen B, Young D, Cole ST (1992). The catalase-peroxidase gene and isoniazid resistance of *Mycobacterium tuberculosis*.. Nature.

[9] Banerjee A, Dubnau E, Quemard A, Balasubramanian V, Um KS (1994). *inhA*, a gene encoding a target for isoniazid and ethionamide in *Mycobacterium tuberculosis*.. Science.

[10] Vilchèze C, Weisbrod TR, Chen B, Kremer L, Hazbon MH (2005). Altered NADH/NAD+ ratio mediates coresistance to isoniazid and ethionamide in mycobacteria.. Antimicrob Agents Chemother.

[11] Telenti A, Imboden P, Marchesi F, Lowrie D, Cole S (1993). Detection of rifampicin-resistance mutations in *Mycobacterium tuberculosis*.. Lancet.

[12] Telenti A, Philipp WJ, Sreevatsan S, Bernasconi C, Stockbauer KE (1997). The *emb* operon, a gene cluster of *Mycobacterium tuberculosis* involved in resistance to ethambutol.. Nat Med.

[13] Scorpio A, Zhang Y (1996). Mutations in *pncA*, a gene encoding pyrazinamidase/nicotinamidase, cause resistance to the antituberculous drug pyrazinamide in tubercle bacillus.. Nat Med.

[14] Finken M, Kirschner P, Meier A, Wrede A, Bottger EC (1993). Molecular basis of streptomycin resistance in *Mycobacterium tuberculosis*: alterations of the ribosomal protein S12 gene and point mutations within a functional 16S ribosomal RNA pseudoknot.. Mol Microbiol.

[15] Caminero JA, Sotgiu G, Zumla A, Migliori GB (2010). Best drug treatment for multidrug-resistant and extensively drug-resistant tuberculosis.. Lancet Infect Dis.

[16] Reynolds MG (2000). Compensatory evolution in rifampin-resistant *Escherichia coli*.. Genetics.

[17] Billington OJ, McHugh TD, Gillepie SH (1999). Physiological cost of rifampin resistance induced in vitro in *Mycobacterium tuberculosis*.. Antimicrob Agents Chemother.

[18] Mariam DH, Mengistu Y, Hoffner SH, Andersson DI (2004). Effect of *rpoB* mutations conferring rifampin resistance on fitness of *Mycobacterium tuberculosis*.. Antimicrob Agents Chemother.

[19] Gagneux S, Long CD, Small PM, Van T, Schoolnik GK (2006). The competitive cost of antibiotic resistance in *Mycobacterium tuberculosis*.. Science.

[20] Björkman J, Hughes D, Andersson DI (1998). Virulence of antibiotic-resistant *Salmonella typhimurium*.. Proc Natl Acad Sci U S A.

[21] Björkman J, Nagaev I, Berg OG, Hughes D, Andersson DI (2000). Effects of environment on compensatory mutations to ameliorate costs of antibiotic resistance.. Science.

[22] vanSoolingen D, de Haas PE, van Doorn HR, Kuijper E, Rinder H (2000). Mutations at amino acid position 315 of the *katG* gene are associated with high-level resistance to isoniazid, other drug resistance, and successful transmission of *Mycobacterium tuberculosis* in the Netherlands.. J Infect Dis.

[23] Pym AS, Saint-Joanis B, Cole ST (2002). Effect of *katG* mutations on the virulence of *Mycobacterium tuberculosis* and the implication for transmission in humans.. Infect Immun.

[24] Sander P, Springer B, Prammananan T, Strumfels A, Kappler M (2002). Fitness cost of chromosomal drug resistance-conferring mutations.. Antimicrob Agents Chemother.

[25] Böttger EC, Springer B (2008). Tuberculosis: drug resistance, fitness, and strategies for global control.. Eur J Pediatr.

[26] Böttger EC, Springer B, Pletschette M, Sander P (1998). Fitness of antibiotic-resistant microorganisms and compensatory mutations.. Nat Med.

[27] Lemey P, Rambaut A, Pybus OG (2006). HIV evolutionary dynamics within and among hosts.. AIDS Rev.

[28] Quiñones-Mateu ME, Arts EJ (2006). Virus fitness: concept, quantification, and application to HIV population dynamics.. Curr Top MicrobiolImmunol.

[29] Oliver A, Canton R, Campo P, Baquero F, Blazquez J (2000). High frequency of hypermutable *Pseudomonas aeruginosa* in cystic fibrosis lung infection.. Science.

[30] Wilder CN, Allada G, Schuster M (2009). Instantaneous within-patient diversity of *Pseudomonas aeruginosa* quorum-sensing populations from cystic fibrosis lung infections.. Infect Immun.

[31] Israel DA, Salama N, Krishna U, Rieger UM, Atherton JC (2001). *Helicobacter pylori* genetic diversity within the gastric niche of a single human host.. Proc Natl Acad Sci U S A.

[32] Suerbaum S, Josenhans C (2007). *Helicobacter pylori* evolution and phenotypic diversification in a changing host.. Nat Rev Microbiol.

[33] Glynn JR, Whiteley J, Bifani PJ, Kremer K, van Soolingen D (2002). Worldwide occurrence of Beijing/W strains of *Mycobacterium tuberculosis*: a systematic review.. Emerg Infect Dis.

[34] Bifani PJ, Mathema B, Kurepina NE, Kreiswirth BN (2002). Global dissemination of the *Mycobacterium tuberculosis* W-Beijing family strains.. Trends Microbiol.

[35] Kremer K, Glynn JR, Lillebaek T, Niemann S, Kurepina NE (2004). Definition of the Beijing/W Lineage of *Mycobacterium tuberculosis* on the Basis of genetic markers.. J Clin Microbiol.

[36] Kamerbeek J, Schouls L, Kolk A, van Agtervveld M, van Soolingen D (1997). Simultaneous detection and strain differentiation of *Mycobacterium tuberculosis* for diagnosis and epidemiology.. J Clin Microbiol.

[37] vanEmbden JDA, Cave MD, Crawford JT, Dale JW, Eisenach KD (1993). Strain identification of *Mycobacterium tuberculosis* by DNA fingerprinting: recommendations for a standardized methodology.. J Clin Microbiol.

[38] Heym B, Alzari PM, Honore N, Cole ST (1995). Missense mutations in the catalase-peroxidase gene, *katG*, are associated with isoniazid resistance in *Mycobacterium tuberculosis*.. Mol Microbiol.

[39] vanDoorn HR, Kuijper EJ, van derEnde A, Welten AGA, van Soolingen D (2001). The susceptibility of *Mycobacterium tuberculosis* to isoniazid and the Arg→Leu mutation at codon 463 of *katG* are not associated.. J Clin Microbiol.

[40] Ramaswamy SV, Amin AG, Goksel S, Stager CE, Dou S-J (2000). Molecular genetic analysis of nucleotide polymorphisms associated with ethambutol resistance in human isolates of *Mycobacterium tuberculosis*.. Antimicrob Agents Chemother.

[41] Goude R, Amin AG, Chatterjee D, Parish T (2009). The arabinosyltransferase EmbC is inhibited by ethambutol in *Mycobacterium tuberculosis*.. Antimicrob Agents Chemother.

[42] Prammananan T, Sander P, Brown BA, Frischkorn K, Onyi GO (1998). A single 16S ribosomal RNA substitution is responsible for resistance to amikacin and other 2-deoxystreptamine aminoglycosides in *Mycobacterium abscessus* and *Mycobacterium chelonae*.. J Infect Dis.

[43] Alangaden GJ, Kreiswirth BN, Aouad A, Khetarpal M, Igno FR (1998). Mechanism of resistance to amikacin and kanamycin in *Mycobacterium tuberculosis*.. Antimicrob Agents Chemother.

[44] Melancon P, Lemieux C, Brakier-Gingras L (1988). A mutation in the 530 loop of *Escherichia coli* 16S ribosomal RNA causes resistance to streptomycin.. Nucl Acids Res.

[45] Shcherbakov D, Akbergenov R, Matt T, Sander P, Andersson DI (2010). Directed mutagenesis of *Mycobacterium smegmatis* 16S rRNA to reconstruct the in-vivo evolution of aminoglycoside resistance in *Mycobacterium tuberculosis*.. Mol Microbiol.

[46] Meacci F, Orru G, Iona E, Giannoni F, Piersimoni C (2005). Drug resistance evolution of a *Mycobacterium tuberculosis* strain from a noncompliant patient.. J Clin Microbiol.

[47] Kaplan G, Post FA, Moreira AL, Wainwright H, Kreiswirth BN (2003). *Mycobacterium tuberculosis* growth at the cavity surface: a microenvironment with failed immunity.. Infect Immun.

[48] Nagaev I, Björkman J, Andersson DI, Hughes D (2001). Biological cost and compensatory evolution in fusidic acid-resistant *Staphylococcus aureus*.. Mol Microbiol.

[49] Maisnier-Patin S, Berg OG, Liljas L, Andersson DI (2002). Compensatory adaptation to the deleterious effect of antibiotic resistance in *Salmonella typhimurium*.. Mol Microbiol.

[50] Sherman DR, Mdluli K, Hickey MJ, Arian TM, Morris SL (1996). Compensatory *ahpC* gene expression in isoniazid-resistant *Mycobacterium tuberculosis*.. Science.

[51] Björkholm B, Sjölund M, Falk PG, Berg OG, Engstrand L (2001). Mutation frequency and biological cost of antibiotic resistance in *Helicobacter pylori*.. Proc NatlAcadSci U S A.

[52] Hurdle JG, O'Neill AJ, Ingham E, Fishwick C, Chopra I (2004). Analysis of mupirocin resistance and fitness in *Staphylococcus aureus* by molecular genetic and structural modeling techniques.. Antimicrob Agents Chemother.

[53] vanRie A, Victor TC, Richardson M, Johnson R, van derSpuy GD (2005). Reinfection and mixed infection cause changing *Mycobacterium tuberculosis* drug-resistance patterns.. Am J Respir Crit Care Med.

[54] Shamputa IC, Jugheli L, Sadradze N, Willery E, Portaels F (2006). Mixed infection and clonal representativeness of a single sputum sample in tuberculosis patients from a penitentiary hospital in Georgia.. Respir Res.

