# Supplementary material for: Dynamics of Antibiotic Resistant Mycobacterium tuberculosis during Long-Term Infection and Antibiotic Treatment
Source: PLoS One. 2011 Jun 16;6(6):e21147. doi: 10.1371/journal.pone.0021147 (PMC3116863; doi:10.1371/journal.pone.0021147)
Supplement: Figure S1 — Spoligotyping and RFLP pattern of serial clinical isolates of M. tuberculosis. S99-293 = isolate 8, S91-222 = isolate 1, S91-224 = isolate 2, S91-263 = isolate 3, S92-001 = isolate 4, S92-031 = isolate 5, S93-007 = isolate 6, S93-021 = isolate 7. (DOCX) [file pone.0021147.s001.docx]

Figure S1. Spoligotyping and RFLP pattern of serial clinical isolates of *M. tuberculosis*. S99-293 = isolate 8, S91-222 = isolate 1, S91-224 = isolate 2, S91-263 = isolate 3, S92-001 = isolate 4, S92-031 = isolate 5, S93-007 = isolate 6, S93-021 = isolate 7.
