# Supplementary material for: Dynamics of Antibiotic Resistant Mycobacterium tuberculosis during Long-Term Infection and Antibiotic Treatment
Source: PLoS One. 2011 Jun 16;6(6):e21147. doi: 10.1371/journal.pone.0021147 (PMC3116863; doi:10.1371/journal.pone.0021147)
Supplement: Table S1 — Primers and PCR conditions for amplification of resistance genes. (DOCX) [file pone.0021147.s002.docx]

Table S1. Primers and PCR conditions for amplification of resistance genes.

| Gene name | Primer pairs | Fragment  length (bp) | Cycling conditions  (for all genes 35 cycles were used) |
| --- | --- | --- | --- |
| *katG* | 5'ATGGGGCTGATCTACGTGAA3'  5'TCCTTGGCGGTGTATTGC3' | 410 | 95^o^C 5min; 94^o^C 30s, 55^o^C 30s, 72^o^C 30s, 72^o^C 7min. |
|  | 5'TACGAGTGGGAGCTGACGAA3'  5'ATGCGGTCGAAACTAGCTGT3' | 429 |  |
| *rpoB* | 5'ATCAACATCCGGCCGGTGGT3'  5' TACACCGACAGCGAGCCGAT3' | 258 | 95^o^C 5min; 94^o^C 20s, 55^o^C 20s, 72^o^C 30s, 72^o^C 7min. |
| *rpsL* | 5'ATGCCAACCATCCAGCAG3'  5'TTCTCTTTCTTAGCGCCGTA3' | 368 | 95^o^C 5min; 94^o^C 30s, 55^o^C 30s, 72^o^C 30s, 72^o^C 7min. |
| *embB* | 5'TGATATTCGGCTTCCTGCTCT3'  5'TTGTTGAACGGCATCCAC 3' | 359 | 95^o^C 5min; 94^o^C 30s, 55^o^C 30s, 72^o^C 30s, 72^o^C 7min. |
|  | 5'TGGATGCCGTTCAACAAC 3'  5'TTCTCGGTATACCACGCCTG3' | 385 | 95^o^C 5min; 94^o^C 30s, 55^o^C 30s, 72^o^C 30s, 72^o^C 7min. |
| *rrs* | 5'GAAACTGGGTCTAATACCGGA3' 5'AAGGAAGGAAACCCACACCTA3' | 688 | 95^o^C 5min; 94^o^C 30s, 55^o^C 30s, 72^o^C 50s, 72^o^C 7min. |
|  | 5'TAGGTGTGGGTTTCCTTCCTT3'  5'ATCCCACCTTCGACAGCTC3' | 660 |  |
